# TDP-43 Identified from a Genome Wide RNAi Screen for SOD1 Regulators

**DOI:** 10.1371/journal.pone.0035818

**Published:** 2012-04-26

**Authors:** Balajee R. Somalinga, Cameron E. Day, Shuguang Wei, Michael G. Roth, Philip J. Thomas

**Affiliations:** 1 Department of Physiology, University of Texas Southwestern Medical Center, Dallas, Texas, United States of America; 2 Department of Biochemistry, University of Texas Southwestern Medical Center, Dallas, Texas, United States of America; National Institutes of Health, United States of America

## Abstract

Amyotrophic Lateral Sclerosis (ALS) is a late-onset, progressive neurodegenerative disease affecting motor neurons in the brain stem and spinal cord leading to loss of voluntary muscular function and ultimately, death due to respiratory failure. A subset of ALS cases are familial and associated with mutations in superoxide dismutase 1 (SOD1) that destabilize the protein and predispose it to aggregation. In spite of the fact that sporadic and familial forms of ALS share many common patho-physiological features, the mechanistic relationship between SOD1-associated and sporadic forms of the disease if any, is not well understood. To better understand any molecular connections, a cell-based protein folding assay was employed to screen a whole genome RNAi library for genes that regulate levels of soluble SOD1. Statistically significant hits that modulate SOD1 levels, when analyzed by pathway analysis revealed a highly ranked network containing TAR DNA binging protein (TDP-43), a major component of aggregates characteristic of sporadic ALS. Biochemical experiments confirmed the action of TDP-43 on SOD1. These results highlight an unexpected relationship between TDP-43 and SOD1 which may have implications in disease pathogenesis.

## Introduction

Amyotrophic lateral sclerosis (ALS) is a progressive neurodegenerative disease that affects motor neurons in the brain stem and spinal cord. Patients afflicted with severe forms of the disease have a median survival time of less than 2 years after the appearance of the symptoms [1], [2]. The loss of motor neurons leads to defects in voluntary muscular activities such as breathing, walking, swallowing *etc.* and fatality occurs usually due to respiratory failure. Aggregated proteinaceous inclusions have been found in the cell bodies of motor neurons derived from patients and mouse models [2], [3]. The aggregates can contain a variety of ubiquitinated proteins including TAR DNA binding protein (TDP-43) or superoxide dismutase 1 (SOD1) [2], [4], [5].

SOD1 is a detoxification enzyme, that catalyzes the conversion of superoxide to hydrogen peroxide [6]. Mutations in SOD1 constitute a significant share (∼20%) of all familial ALS cases [2]. These mutations destabilize SOD1 and promote aggregate formation [7]. TDP-43 is a RNA-DNA binding protein reported to be involved in transcription, splicing and RNA stability [8]. Recent studies suggest that TDP-43 self-regulates its own levels by altering the splicing of its transcripts [9]. TDP-43 aggregates are found in patients with sporadic disease and in most familial versions, excepting SOD1-linked ALS [4]. A subset of familial ALS is associated with mutations in TDP-43 that promote its aggregation [10]. In addition to SOD1 and TDP-43, mutations in several other proteins including progranulin, alsin, senataxin have been associated with fALS. None of these proteins have demonstrated molecular connections.

Regardless of the proteins present in the aggregates, sporadic and familial ALS cases share many patho-physiological characteristics, including inclusion formation, vacuolization of the cell bodies, oxidative damage, motor neuron loss and attendant physiological symptoms [11], suggesting that common molecular processes may lead to the disease phenotype. Unfortunately there is currently a dearth of knowledge about molecular mechanisms that link the patho-physiology of the various sporadic and familial forms of the disease. Some reports suggest that sporadic ALS and SOD1 linked ALS occur due to completely independent mechanisms [12]. To test this hypothesis and to potentially reveal putative underlying molecular connections between the SOD1-linked familial ALS and the other proteins implicated in the disease, an RNAi screen for proteins that regulate soluble levels of SOD1 was performed.

An extant reporter assay [13], [14] that monitors the solubility of proteins in cells was utilized to screen a genome-wide RNAi library for cellular modulators that affect mutant SOD1 solubility and folding. The assay is based on the structural complementation of the two β-galactosidase fragments to form an active enzyme in cells, which can be monitored. The reporter assay consists of SOD1 protein fused to a small fragment (α) of the β-galactosidase enzyme, which is co-expressed with the larger ω fragment. Changes in the soluble levels of mutant SOD1 are linked to availability of the α fragment and are reflected by a change in the assay signal. Thus, knocking down genes from the whole genome may alter the signal, up or down, depending upon their effect on SOD1 solubility, transcription, translation, protein stability or degradation. The hits from the screen were analyzed using pathway analysis software, which identified a network involved in Skeletal and Muscular System Development and Function, Tissue Morphology and Inflammatory Response. Among the hits represented in the network was TDP-43, which dramatically increased the SOD1 assay signal upon knockdown. Validation experiments with TDP-43 knockdown and overexpression confirmed the regulatory role of TDP-43 on SOD1. These findings suggest that this TDP-43 and SOD1 connection provides a link between familial and TDP-43 linked sporadic ALS.

## Results

### Assay development

An assay that monitors the levels of soluble protein inside cells was used in this study [13], [14]. The assay relies upon structural complementation between mutant SOD1 fused with the α fragment of β-galactosidase and the ω fragment of β-galactosidase to regenerate enzymatic activity [15], which can then be measured using a fluorogenic or luminogenic substrate (Figure S1). A4VSOD1-HA-α fusion expression was driven by a minimal human SOD1 promoter [16], [17] and ω fragment expression driven by a CMV promoter. When these reporter plasmids were co-expressed in mammalian cells, the amount of β-gal activity observed was directly correlated to the amount of soluble SOD1 present in cells [14]. Any cellular process that affects the levels of SOD1 will be reflected in the assay signal. For instance, when proteasome activity was inhibited with MG 132, the levels of the SOD1 fusion increased as observed by western blot, resulting in increased assay signal [14].

**Figure 1:**
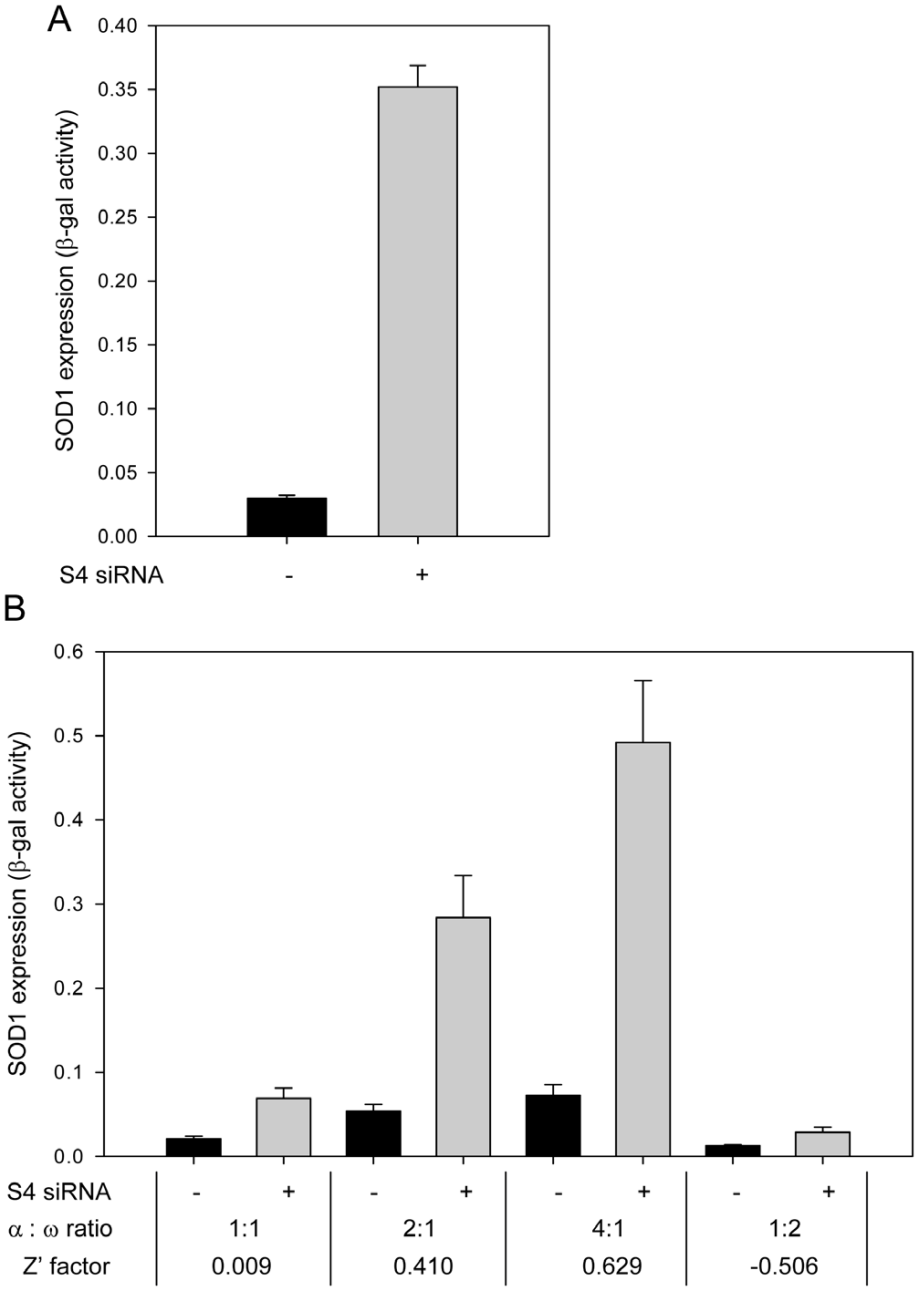
Optimization of the reporter assay for high throughput format. **A**) Effect of knock down of a proteasomal subunit on SOD1 levels. HeLa cells were transfected with siRNA targeting the S4 subunit (PSMC1) of the 26 S proteasome, followed by expression of the mutant SOD1 reporter plasmids. Supernatant fractions were tested for β-gal activity by measuring the fluorescence signal. The plot shows SOD1 levels as a measure of β-gal activity. **B**) HeLa cells were co-transfected with S4 siRNA or carrier and varying amounts of plasmids coding for A4VSOD1-HA-α fusion with a fixed amount of ω fragment. Cells were analyzed for fluorescence 96 hrs post transfection. The SOD1 levels measured as a function of β-gal activity are plotted against each transfection condition. Z′ factor was used to assess the assays suitability for HTS.

A similar effect would be expected upon knockdown of the components of proteasome. siRNA targeted against the S4 (PSMC1) subunit of 26 S proteasome was transfected into HeLa TetOn cells expressing the components of the reporter assay. Assessment of the β-gal activity (soluble SOD1 levels) revealed an increase in fluorescence signal in the samples treated with S4 siRNA (Figure 1A). SOD1 protein levels were found to be elevated when tested in a western blot analysis with probes detecting SOD1 (data not shown). To establish that the observed effect on SOD1 levels was due to the depletion of the S4 subunit of proteasome, samples were analyzed by immunoblot with anti S4 antibody. The results showed >90% knockdown of the S4 protein (data not shown). These results confirmed that proteasomal inhibition through siRNA can be used to optimize the assay for high-throughput screening.

Since the whole genome RNAi library was distributed in 96 well plates, the assay was optimized in this format by systematically varying the reporter plasmid ratios, DNA concentration, transfection reagent volume, cell numbers, incubation time, and plate reading conditions [18]. The influence of one such change involving DNA ratios and amounts significantly improved assay quality (Figure 1B). HeLa-TetOn cells pretreated with S4 siRNA or with no siRNA were transfected with reporter plasmids (SOD1 promoter driven A4VSOD1-HA-α and CMV promoter driven ω) in 1∶1, 2∶1, 3∶1 and 4∶1 ratios (α∶ω) and were incubated for 96 hrs prior to analysis of β-gal activity by the addition of a luminogenic substrate. Cells transfected with plasmids in the ratio 4∶1 had β-gal activity in the response range. The Z′ factor of this condition was greater than 0.5, a minimum threshold, thus, indicating the assay is suitable for high throughput screening [19].

### Screening of the whole genome RNAi library

**Figure 2:**
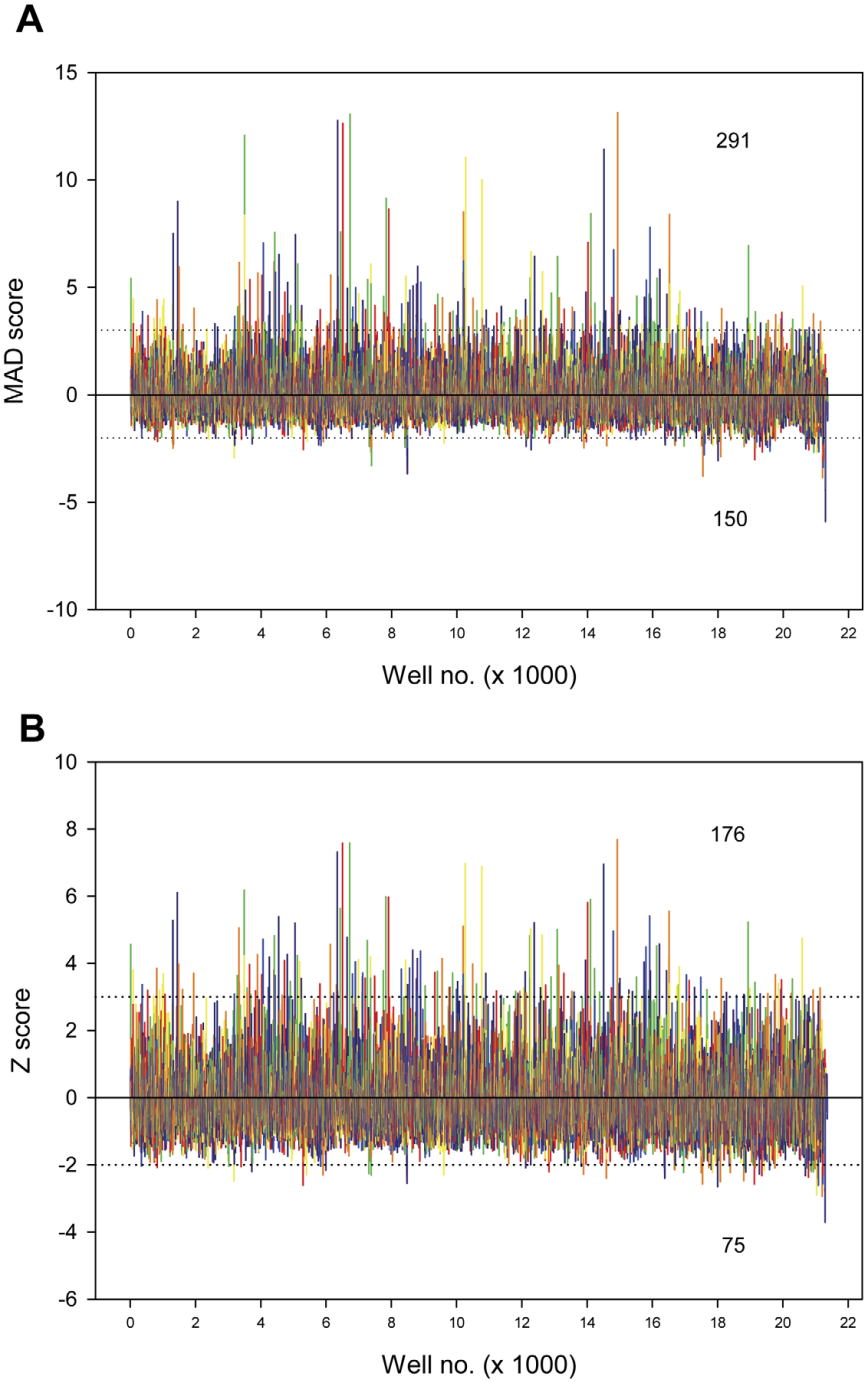
Distribution of β-gal assay signal in the genome wide RNAi screen. **A**) Luminescence intensity measurements of the genome wide RNA screen of 21125 wells in triplicates were processed by MAD score analysis method and the results are plotted against well numbers. Horizontal dotted lines indicate the hit selection criteria of +3 MAD or −2MAD. The two numbers listed in the plot represent the total number of hits that meet the two selection criteria. **B**) Luminescence intensity measurements in triplicates were processed by Z score analysis method and the results are plotted against well numbers. Horizontal dotted lines indicate the hit selection criteria of +3 SD or −2SD. The numbers listed in the plot represent the total number of hits that meet the hit selection criteria.

siRNA pools of four oligonucleotides targeting each of 21,125 human proteins were screened under these optimized assay conditions (Figure S2). The results from the screen were analyzed by two different methods; MAD score, a median based method [20] and Z-score, a mean based method. Plots of the screening data processed by the MAD and Z score based methods are shown in Figure 2A and 2B respectively. There is a high degree of correlation between the hits processed by both methods, *viz.*, close to 90% of the hits from the Z-score method were present in the MAD score based hits. The MAD based method is less prone to plate artifacts and outliers and was chosen for further analyses. The normalized raw data was also used to calculate the q*-value [21] for the assessment of false discovery rate (FDR). There were 291 hits selected above 3 MAD and 150 hits below −2 MAD. A relaxed cut-off value of −2 MAD was selected due to a shallow assay floor as the signal reached closer to the background signal. The hits with MAD scores >3MAD and <−2MAD were used for pathway analysis.

### Functional classes of proteins that modulate SOD1 levels

**Figure 3:**
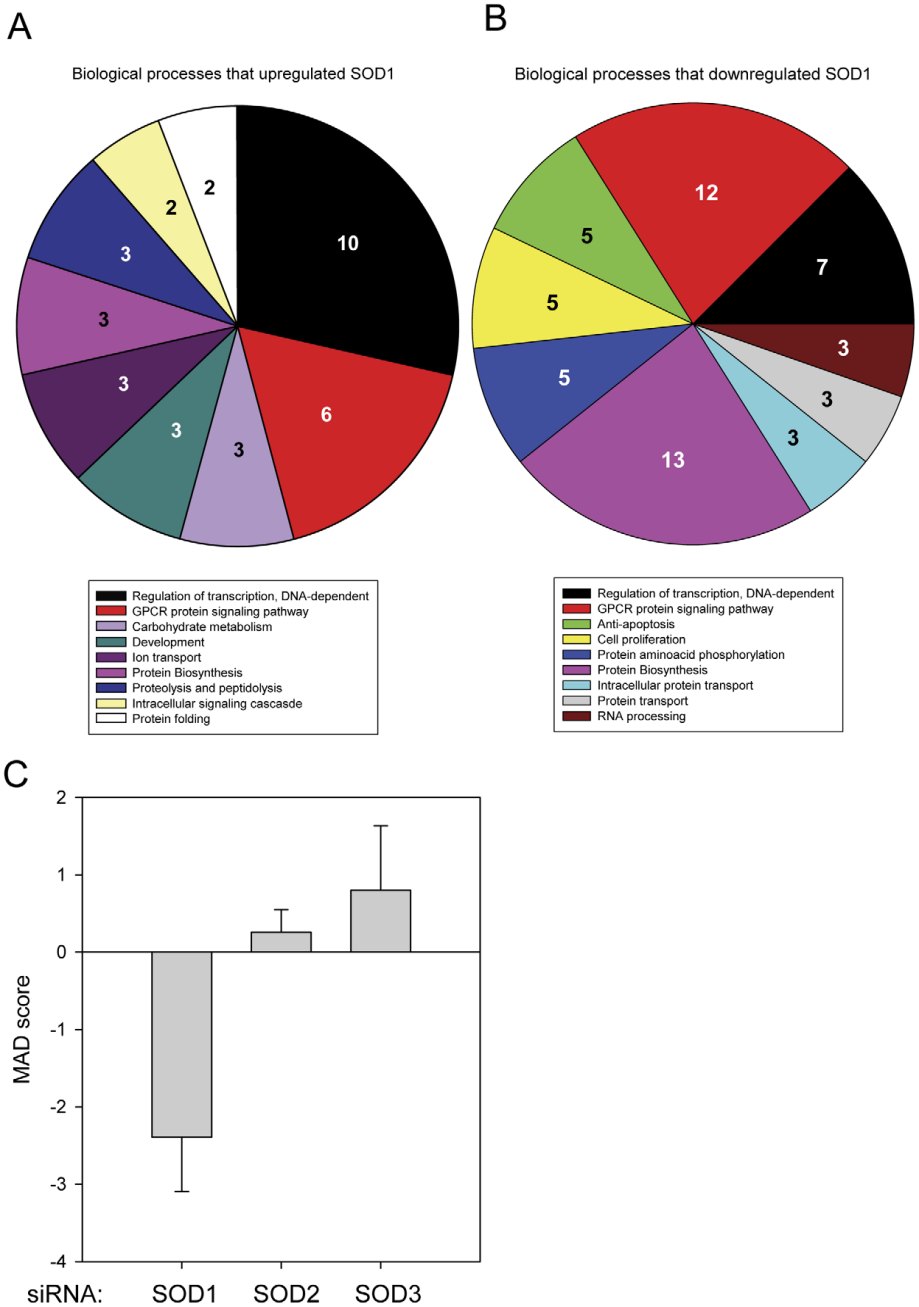
Classification of prominent regulators of SOD1 in the RNAi screen. **A & B**) Pie charts representing annotated biological process of proteins identified as regulators of SOD1. Contribution of each biological process as a percentage of total are shown. Contributions of processes that are less than 2% of the total are not shown in the figure. **C**) Effect of siRNA knockdowns of SOD1 isoforms in the screen. The MAD score values of the three SOD1 isoforms obtained in the screen are shown. The error bars represent SD.

The hits from the siRNA screen that met the selection criteria were classified broadly in to functional categories and a pie chart summarizing the prominent functional processes of the proteins that affected SOD1 levels are shown in Figure 3A&B. Among the targets whose knockdown increased SOD1 levels were proteins involved in regulation of transcription and G-protein coupled receptor signaling. Those whose knockdown decreased SOD1 levels were proteins essential for protein biosynthesis, G-protein coupled receptor signaling and regulation of transcription. The MAD scores calculated for selected targets, such as, SOD isoforms and ribosomal proteins were consistent with expectations. For example, siRNA targeting SOD1 lowered the levels of the reporter levels significantly (−2.4 MAD score) and had a very low q* value (FDR), whereas ones that target SOD2 (0.25 MAD score) or SOD3 protein (0.8 MAD score), had no significant effect (Figure 3C). Knockdown of many proteins that are part of the ribosomal protein machinery, as expected, lowered the levels of reporter signal (data not shown).

### Pathways analysis

**Figure 4:**
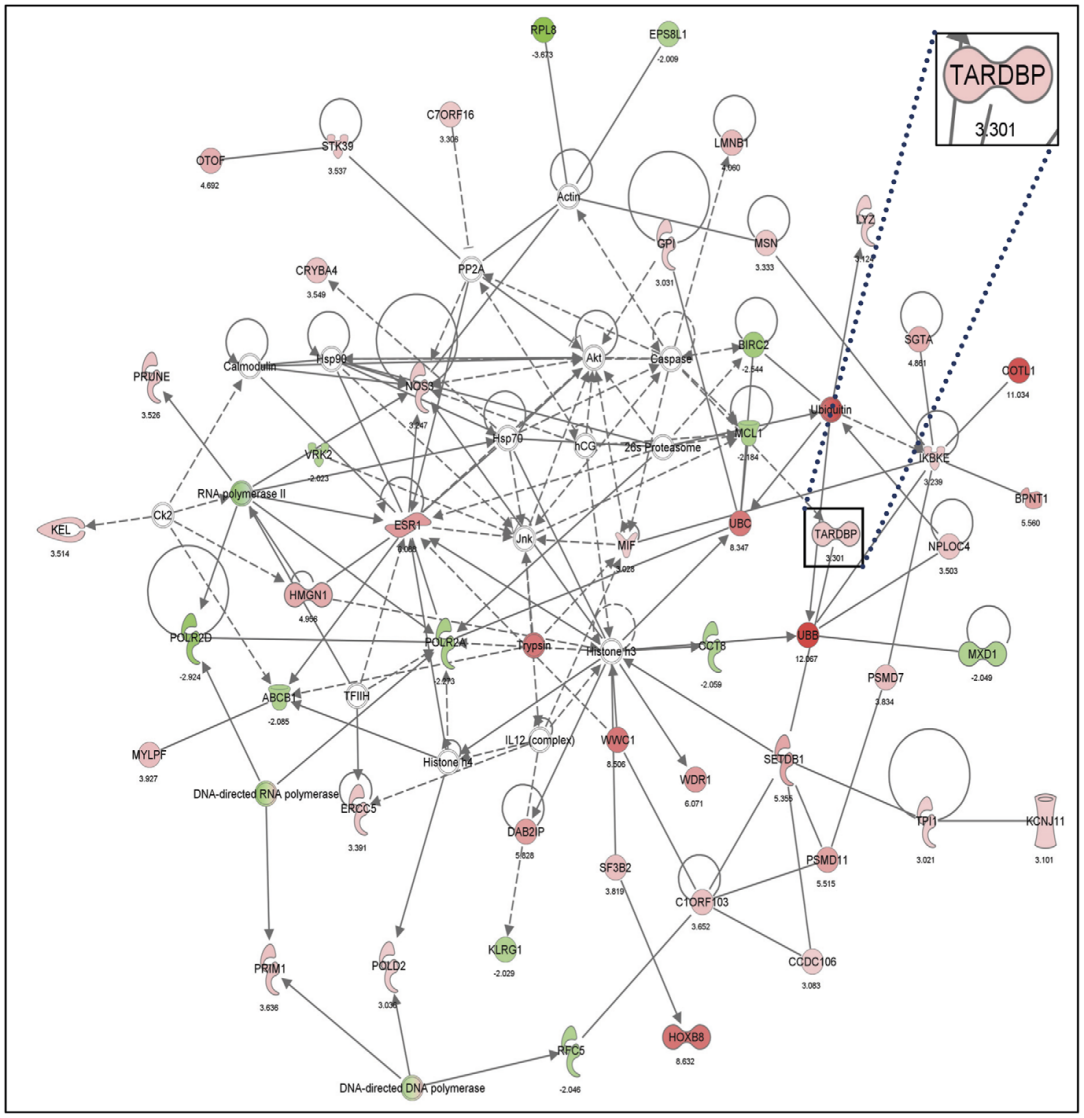
Protein interaction network map of hits from the RNAi screen. The data from the RNAi screen was processed to calculate MAD scores and was used for Ingenuity pathway analysis (IPA). The network annotated “Skeletal and Muscular System Development and Function, Tissue Morphology, Inflammatory Response" ranked at the top with the highest score and is shown in the figure. The hits that increased SOD1 levels above 3SD are shown in red and the ones that decreased the signal below −2SD are shown in green. The proteins represented by color-less nodes were below the selection criteria. Direct interactions are shown in bold lines and indirect interactions are shown in broken lines. (See Table S1 for proteins in this network)

The hits were analyzed for potential relationship with established networks, by Ingenuity pathway analysis (IPA) software. The leading interaction network generated by the IPA program based upon the highest score is shown in Figure 4. The network represents proteins annotated under “Skeletal and Muscular System Development and Function, Tissue Morphology, Inflammatory Response" and contains over 40 proteins that either increased the SOD1 levels >3MAD, represented in red, or that decreased SOD1 levels <−2MAD, represented in green. It is interesting to note that one of the nodes in the interaction network is the TARDBP, Tar DNA binding protein (TDP-43) (Inset in Figure 4). This is a multi-domain RNA, DNA binding protein, reportedly involved in transcriptional regulation, RNA splicing, and other functions [8]. In addition, TDP-43 has been implicated in ALS disease pathogenesis due to its presence in proteinaceous aggregates isolated from sporadic ALS patients and most forms of familial ALS patients, except SOD1-linked forms [4], [5]. The identification of TDP-43 as one of the regulators of SOD1 in an RNAi screen suggested an unexpected link between these proteins.

### Validation of the TDP-43 effect on SOD1

**Figure 5:**
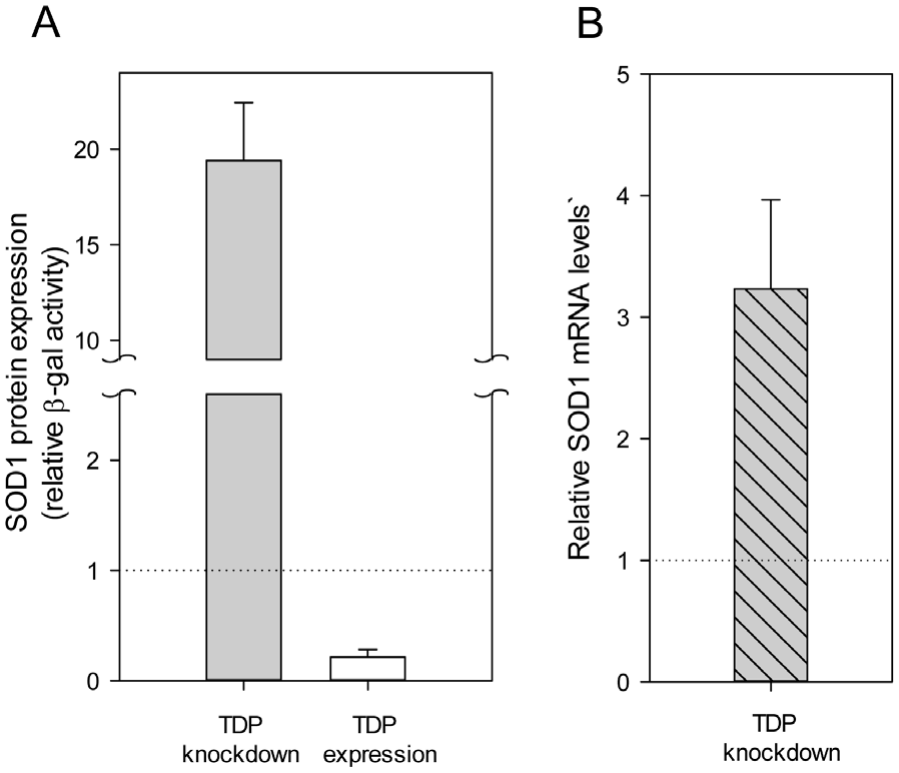
Regulation of SOD1 levels by TDP-43. **A**) TDP-43 knockdown or over expression was carried out in HeLa TetOn cells expressing the A4V reporter plasmids. Supernatant fractions were assayed for β-gal activity. Soluble SOD1 reporter expression levels (β-gal activity) relative to controls (dotted line) are shown. Sample sizes ranged from 8 to 23. The error bars represent 95% confidence interval. **B**) Quantitative PCR analysis of SOD1 reporter mRNA levels was carried out on total mRNA isolated from cells transfected with control or TDP-43 siRNA and A4VSOD1 reporter plasmids. The SOD1 mRNA levels of TDP siRNA treated samples are shown relative to control (dotted line). The error bars represent 95% confidence interval.

In order to confirm the effect of TDP-43 knockdown on SOD1 in the RNAi screen we carried out independent experiments by transfecting HeLa TetOn cells with the TDP siRNA pool followed by monitoring soluble SOD1 levels utilizing the reporter system. Again, upon TDP knockdown, SOD1 levels increased several fold above the control siRNA transfected cells (Figure 5A), confirming the screening results. To validate that the effect was due to TDP-43 and not because of off-target effects of the siRNA, human TDP-43 was over-expressed and SOD1 levels were monitored with the reporter system. In contrast to the increase in SOD1 caused by knockdown, increased TDP-43 expression led to a reduction in SOD1 levels (Figure 5A). The knockdown of TDP-43 and its over expression were confirmed by the anti TDP western blot (Figure S3). These results confirm that TDP-43 can negatively regulate soluble SOD1 levels.

To further validate the screen and the relevance of the TDP-43 linked protein interaction network (Figure 4), ten additional targets identified in the screen (6 targets that increased and 4 targets that decreased SOD1 levels upon knockdown in the screen) were selected. The siGenome siRNA pools targeting the 10 target genes in addition to TDP-43 were independently transfected in to HeLa TetOn cells followed by transfection of the reporter plasmids. The change in SOD1 levels monitored by β-gal assay (Figure S4 A (upper left and right panels) reaffirmed that knockdown of 9 of the 11 targets resulted in statistically significant changes in SOD1 expression. A decrease in the corresponding target message levels upon knockdown with each siRNA pool was confirmed by quantitative PCR (qPCR) analysis (Figure S4 A, lower panel). To evaluate off-target effects of the siRNA pools, experiments were carried out with independent siRNA (On Target Plus) designed against regions of the target proteins distinct from those utilized by the pooled siGenome siRNAs. Five of the nine targets, including TDP43, (Figure S4 B, left panel) exhibited statistically significant changes in the SOD1 levels (measured by β-gal assay), in a direction consistent with that observed in the screen. To verify that the On Target plus siRNAs reduced the target message levels, qPCR was carried on these 5 samples. Significant decrease in the messenger RNA levels was observed (Figure S4 B, right panel) for all. In addition, western blot analysis of samples from cells treated with siGenome siRNA pools or On Target plus siRNA showed clear decreases in the protein levels of all 4 targets assessed (Figure S4 C) further supporting the influence of these 5 targets on SOD1 expression.

### TDP-43 regulates SOD1 mRNA level

TDP-43 has been reported to be involved in many cellular functions including DNA/RNA binding, transcriptional repression, and splicing [8], [22]–[26]. Thus, the SOD1 modulating function of TDP-43 observed here could reasonably result in altered mRNA levels. To determine whether the TDP-43 effect altered SOD1 mRNA levels, total RNA isolated from cells treated with TDP-43 siRNA or control siRNA was analyzed by qPCR. SOD1 mRNA levels increased three fold upon TDP-43 knockdown (Figure 5B), which could indicate increased transcription and/or mRNA stability. The results, however, can not be explained by a splicing effect on SOD1 as cDNA used in SOD1 fusion expression construct lacked intronic regions. These results do not preclude a contribution of other mechanisms, such as effects on the protein turnover rates, contributing to the increase in soluble SOD1 observed after TDP-43 knockdown.

Taken together, these results demonstrate an unexpected function for TDP-43 as a regulator of SOD1. In view of these findings, the mislocalization and aggregation of TDP-43 observed in both sporadic and some familial versions of ALS [12] may have functional consequences for SOD1 and predisposition to degeneration.

## Discussion

Most neurodegenerative diseases, including Alzheimers, Parkinson and Amyotrophic lateral sclerosis, have a common underlying etiology in their pathogenesis; a protein(s) misfolds and accumulates in an intracellular or extra cellular environment [27], [28]. Concomitantly there is a series of poorly understood events that lead to degeneration of a selective subset of cells resulting in the loss of function. In ALS, several aberrant regulatory processes have been reported, such as, mitochondrial dysfunction [29], vacuolization [30], glutamate cytotoxicity [31], [32], axonal retrograde transport [33] and oxidative stress [34], [35]. Some of these regulatory pathways have been linked to one another in a sequential manner [35], while there are many that are obviously not connected. In spite of these complexities, there are many common features of disease pathogenesis between sporadic and familial, SOD1-dependent, ALS [11], [36]–[38] suggesting that common, but unknown, molecular mechanisms are at work. Identifying and deciphering these connections is thus vital to understanding the disease and for effectively developing therapies.

In the present work, this issue has been approached by starting from a known attribute; that misfolded mutant SOD1 is linked to familial amyotrophic lateral sclerosis and utilizing a functional genomic approach to identify cellular proteins involved in regulation of this attribute. A cell-based, β-galactosidase assay that monitors SOD1 solubility/folding in cells, [13], [14] was adapted for the whole genome RNAi screening. Earlier studies [14] had demonstrated that blockage of the proteasome by MG132, leads to the elevation of the assay signal due to accumulation of SOD1. However, inhibitors were not suitable for optimizing the assay for an RNAi screen, because of the differences in the treatment procedures. Thus, siRNA targeting S4 (PSMC1), a component of 26 S proteasome was selected and used for assay optimizations (Figure 1A). Screening for modulators of SOD1 solubility/folding was carried out utilizing a whole genome RNAi library containing siRNA pools targeting 21,125 targets, thus providing a near comprehensive scan of genomic proteins. The screen results were processed using two independent statistical methods (MAD score and Z score) minimizing the influence of any anomalies introduced by a particular approach (Figure 2A and 2B). The screen results underrepresented the hits that lowered the level of SOD1 below the typical hit selection threshold of −3SD due to a threshold effect. The range of MAD score values reflects this phenomenon (13 MAD to ∼−6 MAD). Thus, a relaxed hit selection criterion of −2SD was selected to compensate. When the hits that belonged to the selection criteria were classified in to functional categories, it revealed that proteins from the G-protein coupled receptor super family were involved in both up and down-regulation of soluble SOD1 levels (Figure 3A & 3B), demonstrating the complexity of the GPCR signaling network. Further, the occurrence of transcriptional and translational regulatory proteins among the top classes of protein targets involved in up or down regulation of SOD1 was consistent with their role in controlling protein expression levels. In addition, the leading biological processes including GPCR signaling and transcriptional regulation from the RNAi screen, represented many of the cellular modulators of superoxide dismutase 1 identified from an earlier expression cloning screen [14]. Human counterparts of 6 of the 10 murine proteins [14] that were identified in the earlier screen (Table S2) had MAD scores indicating decreased SOD1 expression upon knockdown. Thus, levels of these targets correlate with levels of SOD1; increased expression leads to increased SOD1 and decreased expression, reduces SOD1.

Pathway analyses of the hits from the RNAi screen revealed many protein interaction networks. The network named “Skeletal and Muscular System Development and Function, Tissue Morphology, Inflammatory Response" with the highest score contained over 40 proteins that met the hit criteria (Figure 4). A protein of particular interest in the network because of its relationship to the ALS was the TAR DNA binding protein or TDP-43. TDP-43 is a 414 amino acid, 43 kDa protein that regulates gene expression at the level of transcription, splicing and mRNA stability [39]. Aggregates of TDP-43, including phosphorylated and truncated forms are found in sporadic and many familial forms of ALS [40]. The molecular mechanism(s) by which misfolding of TDP-43 or formation of its aggregates lead to neurodegeneration is poorly understood. A linkage between TDP-43 and SOD1 is suggested not only by the related pathology, but also because of apparent reciprocal relationship of the two proteins in inclusions [26]. TDP-43 aggregates are not observed in familial SOD1-associated ALS patients that harbor SOD1 inclusions. However, it should be noted that TDP-43 has been found to be mis-located to the cytosol [41], [42] in some of these patients. It has also been hypothesized that SOD1 dependent and independent ALS (sporadic and other forms of familial ALS) arise from independent mechanisms [12].

In addition to TDP-43 protein, other TDP-43 associated gene products in the top network also significantly affected SOD1 levels upon knockdown, highlighting the relevance of the network in SOD1 regulation (Figure 4, Table S1). In addition SETDB1, a histone methyl transferase linked to TDP-43 in the network based on its interaction identified in a mass-spectrometry based proteomics study [43] significantly affected SOD1 levels (MAD score 5.3) upon its knockdown. SETDB1 knockdown increased the soluble SOD1 levels both in the screen (Figure 4 and Table S1) and in independent experiments (data not shown). Further, RBMX, a hetero-nuclear Ribonucleo protein (hnRNP) identified among the hits in the top network with a MAD score of 9.9, belongs to the same family of RNP's that has been reported to interact with TDP-43 [44]–[46]. These observations further support the involvement of TDP-43 in the regulation of SOD1.

Validation experiments confirm that the action of TDP-43 on SOD1 observed in the RNAi screen. The lowering of the TDP-43 levels with siRNA treatment resulted in the concomitant increase in SOD1 levels (Figure 5A). A converse experiment showing increased TDP-43 expression resulted in a suppression of SOD1 expression as measured by β-gal activity (Figure 5A). The inverse correlation between increased TDP-43 and decreased SOD1 expression suggests a repressor role for TDP-43. Such a repressor function of TDP-43 on select gene targets has been observed by others [23], [26], [47]. The validation of 5 out of 11 target genes from the TDP-43 linked protein interaction network as regulators of SOD1 expression (Figure S4 A, B) adds further credence to the importance of the network (Figure 4). The current finding that TDP-43 regulates SOD1 is also consistent with a recent micro array study, where a modest increase in SOD1 mRNA levels was observed upon TDP knockdown [48]. The increase in SOD1 reporter mRNA levels as observed by qPCR analysis (Figure 5B) upon TDP-43 knockdown could result because of alterations in transcription or mRNA stability, brought about by TDP-43. However the effect could also be mediated indirectly through an unknown factor which may or may not involve splicing, another known function of TDP-43. As the above experiments were carried out using artificial cDNA SOD1 fusion constructs, such a mechanism would not be observed. Thus, future work will be required to address these possibilities and their importance in the details of the mechanism of TDP-43 repression.

The data presented here (Figure 5A & 5B) revealed an unexpected regulatory link associating TDP-43 and SOD1. Interesting questions for future work will be to determine whether mutant TDP-43 or the aggregated forms of wildtype TDP-43 lose or gain function and how these processes might relate to SOD1 and the dominant toxic-gain-of-function phenotype.

## Materials and Methods

### Plasmids and reagents

The reporter assay plasmids were modified versions of the plasmids used previously [14]. Three different native variants of the human SOD1 promoter have been used in the study. A promoter of length 2156 bp as reported in [16] was used for the RNAi screening. Validation experiments were carried out using reporter plasmids containing a promoter of length 2206 bases with a Thymidine base at position −141 which was cloned from the genomic DNA isolated from HEK293 cells. HEK 293 [49] cells were obtained from ATCC. Site directed mutagenesis of this SOD1 promoter was carried out to generate the G at position −141 to achieve a SOD1 promoter sequence similar to Ensembl listed seq.: ENSG00000142168. Omega fragment of β-galactosidase enzyme was cloned in to pcDNA3.1 vector as mentioned earlier [14]. pBUDCE4.1-TDP-43, a human TDP-43 expression vector provided by Dr. Jeffrey Elliot, was modified with an insertion of a β-lactamase cassette derived from pcDNA3.1 vector. The whole genome human siRNA library consisted of 21,125 pools and was procured from Dharmacon. Each siGENOME Smartpool from the library contained 4 different siRNAs targeting each gene product. On Target plus siRNAs were purchased from Dharmacon. HeLa TetOn [50] (Clontech) cells used in the screening were provided by Dr. Hongtao Yu. Lipofectamine RNAimax was from Invitrogen. Polyethylenimine (PEI), 25 kD, linear polymer was procured from Polysciences and dissolved in water at 1 mg/ml. Validated qPCR primers (Origene) for the targets were used in the study. Mouse anti WWC1 antibody was a kind gift from Dr. Joachim Kremerskothen. Rabbit polyclonal antibodies against COTL1, CCT8 and TDP-43 were purchased from ProteinTech group.

### Whole genome RNAi screening

5.6 µl of each 5 µM stock siRNA Smartpool (Dharmacon) was picked using a Biomek FX (Beckmann coulter) and diluted in to 100 µl of OptiMEM I (Invitrogen) to achieve a concentration of 8 pmol/30 µl. 30 µl of the diluted siRNA was transferred to 96 well assay plates in triplicates. 20 µl of diluted Lipofectamine RNAImax (1∶75 v/v in OptiMEM I) was added using a Microflo liquid dispenser (BioTek) on top of the diluted siRNA pools in assay plates and incubated for 30 minutes at room temperature. Appropriate volume of stock reporter plasmids were diluted in to DMEM (Invitrogen) to generate 200 ng of A4V mutant SOD1-α and 50 ng of ω plasmids in 30 µl volume. Appropriate amounts of PEI to achieve 5 ul/well was added to the diluted DNA, mixed and incubated at room temperature for 15 min. After 15 minutes, HeLa TetOn cells that had been trypsinized and re-suspended in complete DMEM (DMEM+10%FBS+1% Penicillin/Streptomycin) were mixed with the plasmid transfection reaction to obtain a final concentration of 10,000 cells/140 µl. The mixture was incubated at room temperature for 15 minutes. Following incubation, the 140 µl of cell-plasmid transfection mix was added to the ongoing RNAi transfection in the 96 well assay plates using a Titertek liquid dispenser. The 96 well assay plate consisted of columns 1 and 12 dedicated for controls and column 2 through 11 for test siRNA (Figure S2). The assay plates were incubated in a 5% CO_2_/37°C incubator for 96 hrs. Plates were decanted by centrifugation and 50 ul of luminogenic substrate (Beta-glo assay system (Promega) diluted 1∶5 v/v in PBS/0.1% Tween 20) was added to each well. Luminescence measurements were made using a multi label plate reader (Envision, Perkin Elmer). The data obtained were archived for later analyses.

### Data analysis and pathway mapping

Data in triplicates from the 21,125 targets analyzed in the screen were processed by two different methods to verify consistency 1) MAD score and 2) Z score. Z score [51] method uses the mean of the plate to normalize each well data, whereas MAD score uses median absolute deviation [20] of the plate to normalize each well data. They were calculated using the formula: Z score = (Xi−X)/σ_x_, MAD score = (Xi−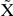)/MAD, where Xi is fluorescent signal in each well, X is the plate mean, σ_x_ is the plate standard deviation, 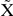 is plate median and MAD is median absolute deviation of the plate. The NCBI accession ID of the targets with varying hit selection criteria were uploaded in to the Ingenuity pathway analysis program and analyzed using the IPA Core analysis module for networks that are highly represented by the hits. Networks with the lowest −log P values indicate a network with the largest number of hits mapped. The hits picked for further analysis were confirmed to meet the false discovery rate q* of <0.05 (FDR was calculated using the Benjamini-Hochberg method [21]

### Monitoring changes in SOD1 level by β-galactosidase reporter assay and Western blot analysis

For knockdown experiments, HeLa TetOn cells were plated in a 6 well plate and grown overnight in an incubator. Cell samples where then transfected with respective siRNA at a concentration of 40 pmol/well using lipofectamine RNAimax according to manufacturers' instructions and incubated for 2 days. After this, cells were transfected with respective reporter systems with PEI and incubated for an additional 2 days. Approximately 96 hrs after siRNA transfection, cells were lysed in 1× reporter lysis buffer (Promega) containing 1 protease inhibitor cocktail mini tablet/10 ml. Cell lysates were spun down for 2 min at 16000 g and total protein content of the supernatant was determined by the Bradford method (Biorad). A volume of each supernatant containing 25 µg of total protein was added with fluorogenic substrate, Fluorescein di-β-D-galactopyranoside (FDG) and the fluorescence was measured (Ex: 490 nm, Em: 525 nm) in a M5 plate reader (Molecular devices). The initial velocity (Vo) of the fluorescence change for the first 11 hrs (linear phase) of the kinetic reaction was used in the calculation. Relative fold change was calculated in reference to control or untreated samples. Volumes containing 25 µg of total protein of the supernatant fraction were loaded on a Tricine SDS-PAGE gel and proteins were transferred to a nitrocellulose membrane and blotted with respective antibodies. The blots were scanned using Typhoon imager (GE Healthcare) and band intensity was quantified whenever required using Image Quant software (GE Healthcare) with Local Averaging used for background correction.

### Quantitative PCR analysis

Total RNA was isolated from cells using the RNAII isolation kit (Clontech) following the manufacturers instructions. The amount of total RNA was evaluated by Broad Range RNA quantification Kit, using Qbit method (Invitrogen). cDNA synthesis from equivalent amounts of the total RNA were performed with the High Capacity cDNA synthesis kit from Applied Biosystems following the manufacturers recommendations. Equivalent volumes of cDNA were then used to set up qPCR reactions of the appropriate target genes and controls using SYBR® GreenER™ qPCR Supermix (Invitrogen) according the manufacturers protocols. qPCR analysis was carried out on an Applied Biosystems 7900HT Fast-Real time PCR system. The fold change in mRNA levels was calculated by the 2 ^−ΔΔCt^ method ([52]).

## Supporting Information

Figure S1 **Assay for soluble, folded SOD1.** The assay system consists of a vector driving expression of SOD1 fused to the α-fragment of β-galactosidase and a separate vector driving expression of the ω-fragment of β-galactosidase. The structural complementation of the α and the ω fragments results in the regeneration of β-galactosidase enzymatic activity that can be measured by a luminogenic substrate. Any changes in biological processes including degradation and aggregation that result in the changes in the soluble levels of the SOD1-fusion will have a concomitant change in luminescence.
(TIF)

Figure S2 **siRNA screening transfection scheme.** Co-transfection scheme of the plasmid and siRNA as carried out in the genome-wide screen. The sample distribution format in a 96 well plate is also shown.
(TIF)

Figure S3 **Changes in TDP-43 protein levels upon TDP-43 knockdown and over expression.** TDP-43 knockdown or over expression was carried out in HeLa TetOn cells expressing the A4V reporter plasmids. Supernatant fractions were run on SDS-PAGE and transferred on to nitrocellulose membrane and blotted with anti-TDP-43 antibody. TDP-43 protein levels (grey bars) quantified from western blots normalized relative to controls (dotted line) are shown.
(TIF)

Figure S4 **Changes in SOD1 expression and target mRNA and protein levels after knockdown of targets in the TDP-43 protein interaction network.** **A**) Relative SOD1 expression as measured by β-gal assay in cells transfected with siGenome siRNA pools targeting 7 targets (*upper left panel*) that increased SOD1 levels in the screen and 4 targets (*upper right panel*) that decreased the SOD1 levels in the screen. All eleven targets are connected in the protein interaction network (Figure 4 and Table S1). Targets that were not statistically significant with P>0.01 in the retest are shown as white bars. Target message levels measured by qPCR in cells transfected with siGenome siRNA pools are shown relative to controls (*lower panel*). **B**) SOD1 protein expression measured by β-gal activity in supernatant fractions of cells transfected with On Target plus siRNA relative to controls is shown (*left panel*). The effects of all five targets were statistically significant (P<0.01). Target message levels measured by qPCR in cells transfected with On Target plus siRNA pools are shown (*right panel*) relative to controls. **C**) Detection of target proteins by western blotting of samples from cells treated with siGenome or On Target Plus siRNA and their respective controls. The error bars in the β-gal assay experiments represent a 95% confidence interval. The error bars in qPCR experiments represent SD.
(TIF)

Table S1 **List of proteins in the interaction network.** Proteins identified in the screen that are involved in the interaction network annotated “Skeletal and Muscular System Development and Function, Tissue Morphology, Inflammatory Response" and represented in **Figure 4**. Gene symbol, NCBI Accession ID and the respective MAD score are shown.
(DOC)

Table S2 **Cellular modulators of Superoxide dismutase 1.** The Gene symbol, equivalent human NCBI Accession ID and MAD scores from the RNAi screen for ten cellular proteins identified in an earlier cDNA expression screen (Table 1 [14]) for gene products whose expression increased soluble SOD1. None of the cellular modulators met the hit selection threshold of the RNAi screen.
(DOC)
