# Supplementary figures and images for: TDP-43 Identified from a Genome Wide RNAi Screen for SOD1 Regulators
Source: PLoS One. 2012 Apr 26;7(4):e35818. doi: 10.1371/journal.pone.0035818 (PMC3338536; doi:10.1371/journal.pone.0035818)

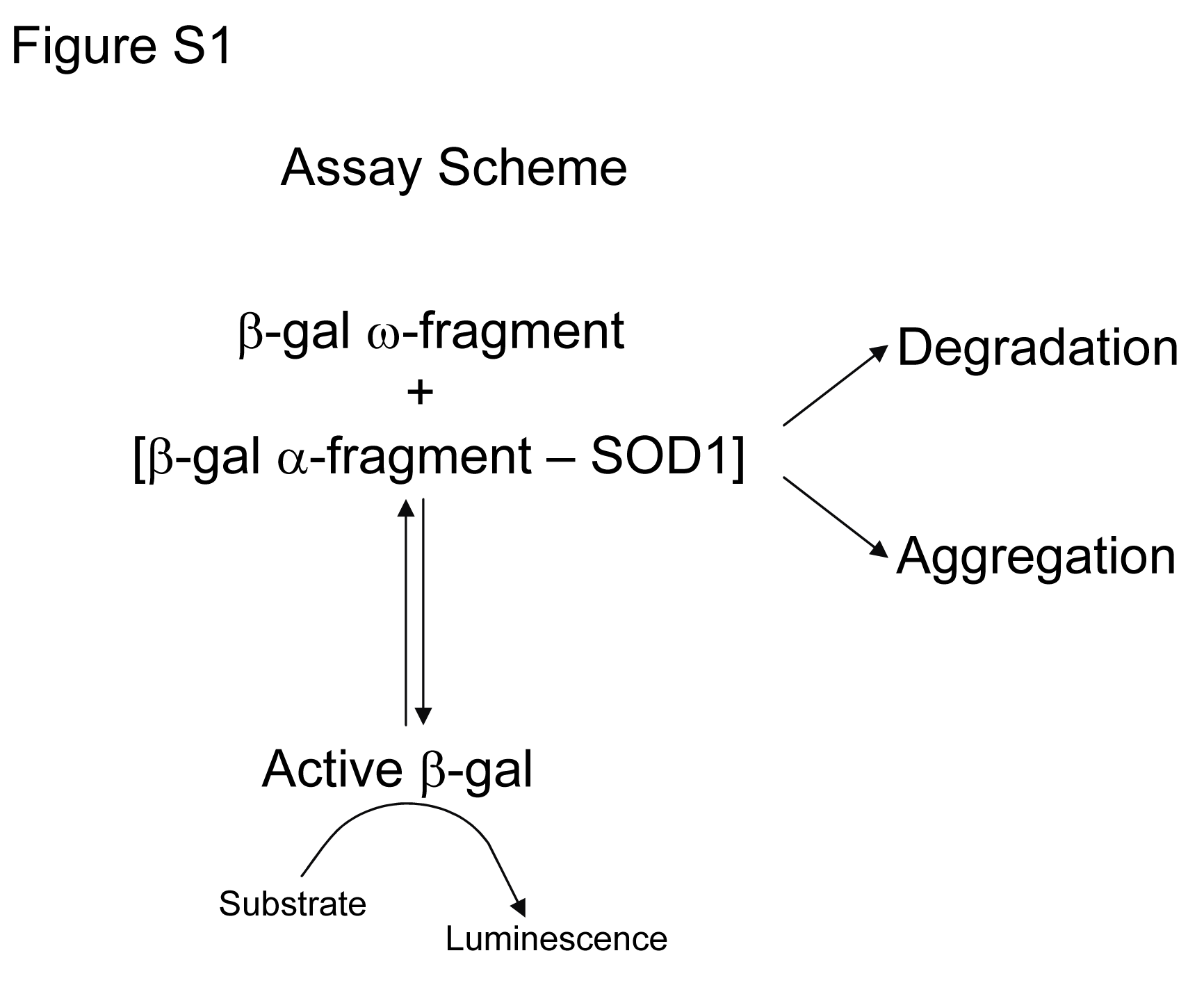

Supplement: Figure S1 — Assay for soluble, folded SOD1. The assay system consists of a vector driving expression of SOD1 fused to the α-fragment of β-galactosidase and a separate vector driving expression of the ω-fragment of β-galactosidase. The structural complementation of the α and the ω fragments results in the regeneration of β-galactosidase enzymatic activity that can be measured by a luminogenic substrate. Any changes in biological processes including degradation and aggregation that result in the changes in the soluble levels of the SOD1-fusion will have a concomitant change in luminescence. (TIF) [file pone.0035818.s001.tif]

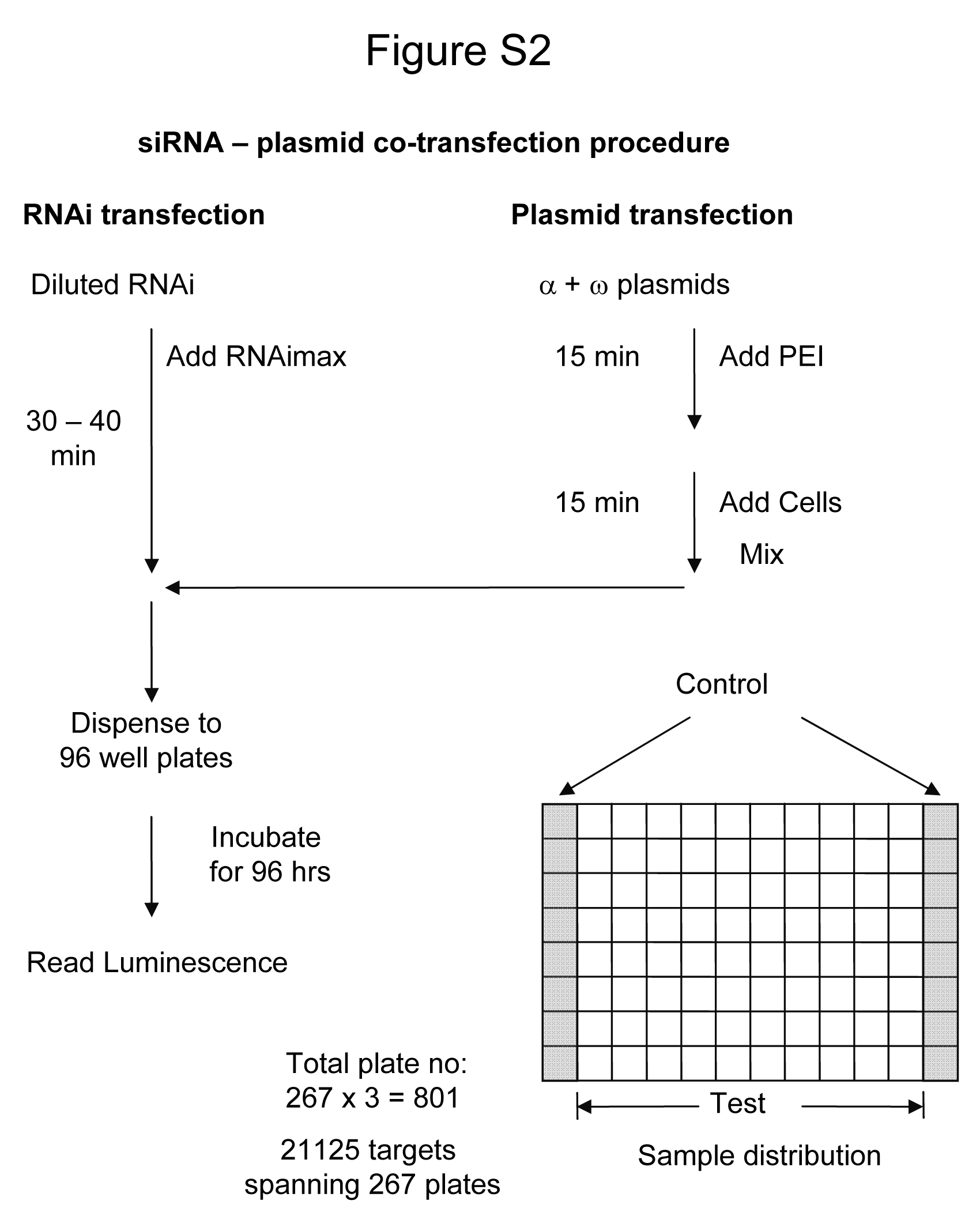

Supplement: Figure S2 — siRNA screening transfection scheme. Co-transfection scheme of the plasmid and siRNA as carried out in the genome-wide screen. The sample distribution format in a 96 well plate is also shown. (TIF) [file pone.0035818.s002.tif]

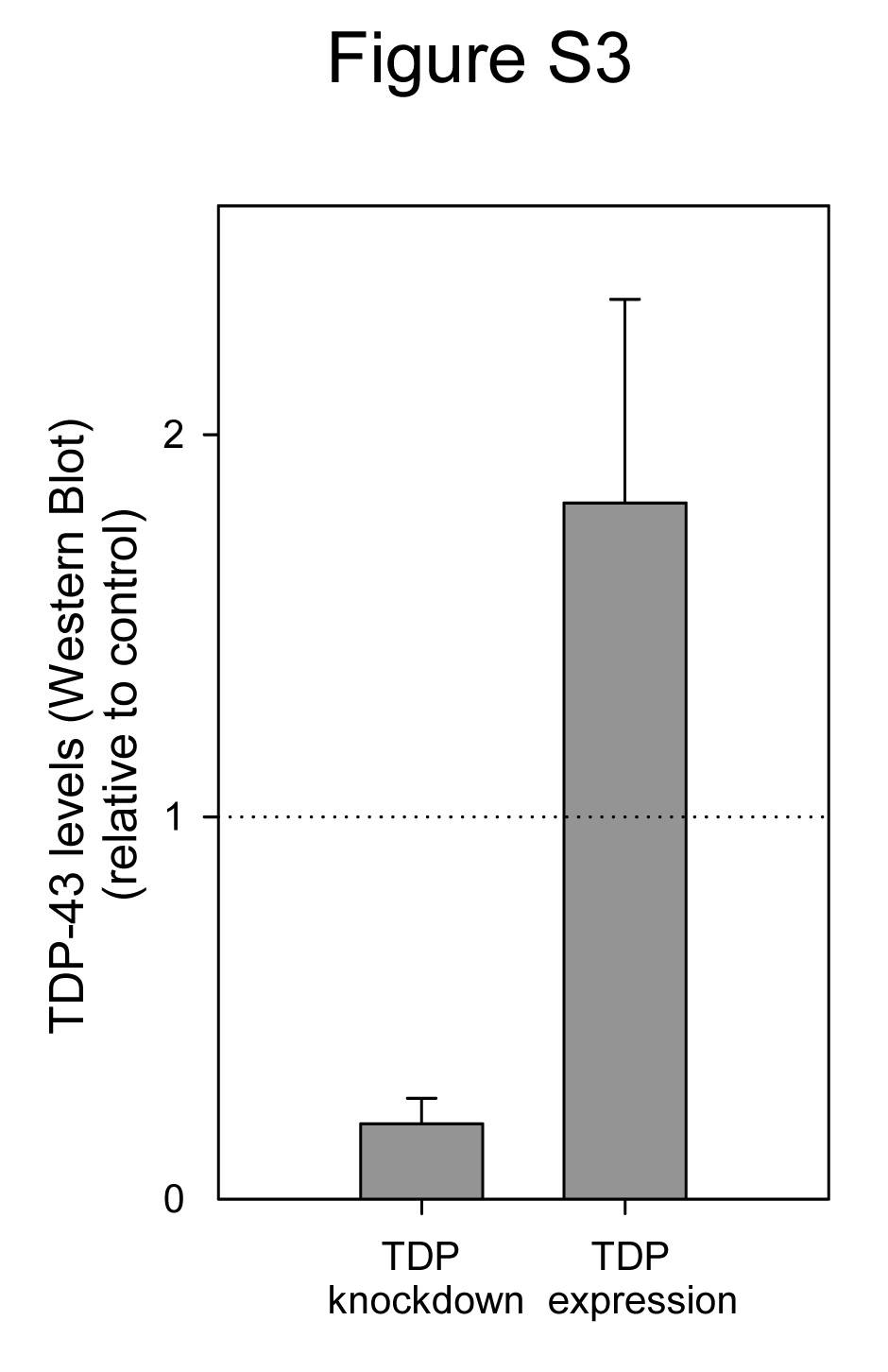

Supplement: Figure S3 — Changes in TDP-43 protein levels upon TDP-43 knockdown and over expression. TDP-43 knockdown or over expression was carried out in HeLa TetOn cells expressing the A4V reporter plasmids. Supernatant fractions were run on SDS-PAGE and transferred on to nitrocellulose membrane and blotted with anti-TDP-43 antibody. TDP-43 protein levels (grey bars) quantified from western blots normalized relative to controls (dotted line) are shown. (TIF) [file pone.0035818.s003.tif]

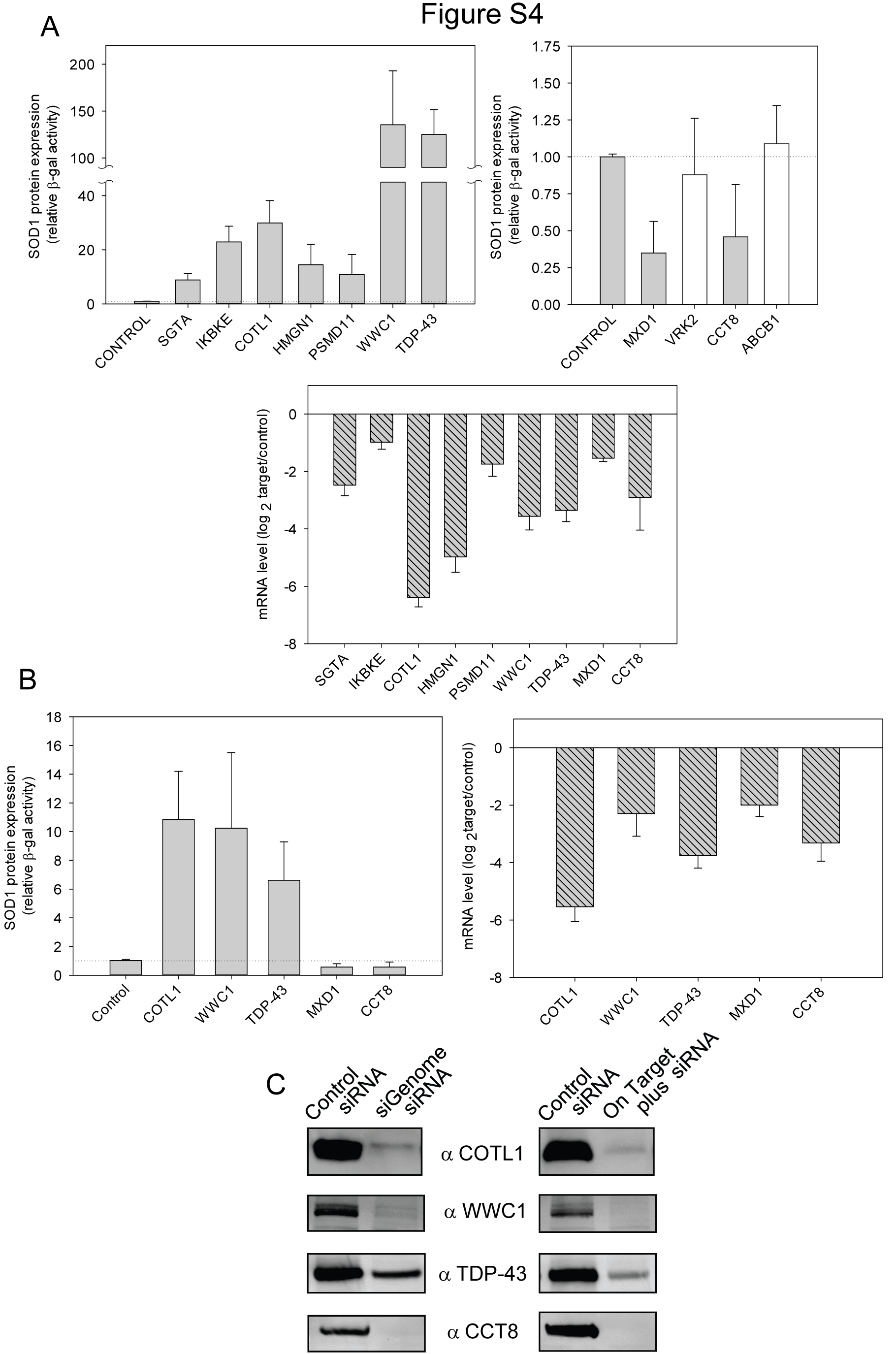

Supplement: Figure S4 — Changes in SOD1 expression and target mRNA and protein levels after knockdown of targets in the TDP-43 protein interaction network. A) Relative SOD1 expression as measured by β-gal assay in cells transfected with siGenome siRNA pools targeting 7 targets (upper left panel) that increased SOD1 levels in the screen and 4 targets (upper right panel) that decreased the SOD1 levels in the screen. All eleven targets are connected in the protein interaction network (Figure 4 and Table S1). Targets that were not statistically significant with P>0.01 in the retest are shown as white bars. Target message levels measured by qPCR in cells transfected with siGenome siRNA pools are shown relative to controls (lower panel). B) SOD1 protein expression measured by β-gal activity in supernatant fractions of cells transfected with On Target plus siRNA relative to controls is shown (left panel). The effects of all five targets were statistically significant (P<0.01). Target message levels measured by qPCR in cells transfected with On Target plus siRNA pools are shown (right panel) relative to controls. C) Detection of target proteins by western blotting of samples from cells treated with siGenome or On Target Plus siRNA and their respective controls. The error bars in the β-gal assay experiments represent a 95% confidence interval. The error bars in qPCR experiments represent SD. (TIF) [file pone.0035818.s004.tif]
