# Supplementary material for: TDP-43 Identified from a Genome Wide RNAi Screen for SOD1 Regulators
Source: PLoS One. 2012 Apr 26;7(4):e35818. doi: 10.1371/journal.pone.0035818 (PMC3338536; doi:10.1371/journal.pone.0035818)
Supplement: Table S1 — List of proteins in the interaction network. Proteins identified in the screen that are involved in the interaction network annotated “Skeletal and Muscular System Development and Function, Tissue Morphology, Inflammatory Response" and represented in Figure 4 . Gene symbol, NCBI Accession ID and the respective MAD score are shown. (DOC) [file pone.0035818.s005.doc]

**Table S**1:

| No. | Gene Symbol | NCBI Accession ID. | MAD score |
| --- | --- | --- | --- |
| 1 | UBB | NM_018955 | 12.067026 |
| 2 | COTL1 | NM_021149 | 11.034039 |
| 3 | HOXB8 | NM_024016 | 8.6320247 |
| 4 | WWC1 | NM_015238 | 8.5060314 |
| 5 | UBC | NM_021009 | 8.3470129 |
| 6 | ESR1 | NM_000125 | 6.0879378 |
| 7 | WDR1 | NM_005112 | 6.0710384 |
| 8 | DAB2IP | NM_032552 | 5.8276935 |
| 9 | BPNT1 | NM_006085 | 5.5604445 |
| 10 | PSMD11 | NM_002815 | 5.5154642 |
| 11 | SETDB1 | NM_012432 | 5.3546111 |
| 12 | HMGN1 | NM_004965 | 4.9562125 |
| 13 | SGTA | NM_003021 | 4.8608834 |
| 14 | OTOF | NM_004802 | 4.6923143 |
| 15 | LMNB1 | NM_005573 | 4.060077 |
| 16 | MYLPF | NM_013292 | 3.9266608 |
| 17 | PSMD7 | NM_002811 | 3.8339004 |
| 18 | SF3B2 | XM_290506 | 3.8189434 |
| 19 | C1ORF103 | NM_018372 | 3.6516202 |
| 20 | PRIM1 | NM_000946 | 3.6357738 |
| 21 | CRYBA4 | NM_001886 | 3.549115 |
| 22 | STK39 | NM_013233 | 3.5371827 |
| 23 | PRUNE | NM_021222 | 3.5262774 |
| 24 | KEL | NM_000420 | 3.5139739 |
| 25 | NPLOC4 | NM_017921 | 3.502967 |
| 26 | ERCC5 | NM_000123 | 3.3914332 |
| 27 | MSN | NM_002444 | 3.3328825 |
| 28 | C7ORF16 | NM_006658 | 3.3057674 |
| 29 | **TARDBP** | **NM_007375** | **3.3007328** |
| 30 | NOS3 | NM_000603 | 3.2472821 |
| 31 | IKBKE | NM_014002 | 3.2391802 |
| 32 | LYZ | NM_000239 | 3.1240945 |
| 33 | KCNJ11 | NM_000525 | 3.1005962 |
| 34 | CCDC106 | NM_013301 | 3.0831603 |
| 35 | POLD2 | NM_006230 | 3.0360999 |
| 36 | GPI | NM_000175 | 3.0307554 |
| 37 | MIF | NM_002415 | 3.0279643 |
| 38 | TPI1 | NM_000365 | 3.0213471 |
| 39 | EPS8L1 | NM_017729 | -2.0086894 |
| 40 | VRK2 | NM_006296 | -2.0232015 |
| 41 | KLRG1 | NM_005810 | -2.0291287 |
| 42 | RFC5 | NM_007370 | -2.0461208 |
| 43 | MXD1 | NM_002357 | -2.0486497 |
| 44 | CCT8 | NM_006585 | -2.058939 |
| 45 | ABCB1 | NM_000927 | -2.08452 |
| 46 | MCL1 | NM_021960 | -2.1840596 |
| 47 | POLR2A | NM_000937 | -2.2730236 |
| 48 | BIRC2 | NM_001166 | -2.543598 |
| 49 | POLR2D | NM_004805 | -2.9244462 |
| 50 | RPL8 | NM_000973 | -3.672558 |
