# Supplementary material for: TDP-43 Identified from a Genome Wide RNAi Screen for SOD1 Regulators
Source: PLoS One. 2012 Apr 26;7(4):e35818. doi: 10.1371/journal.pone.0035818 (PMC3338536; doi:10.1371/journal.pone.0035818)
Supplement: Table S2 — Cellular modulators of Superoxide dismutase 1. The Gene symbol, equivalent human NCBI Accession ID and MAD scores from the RNAi screen for ten cellular proteins identified in an earlier cDNA expression screen (Table 1 [14]) for gene products whose expression increased soluble SOD1. None of the cellular modulators met the hit selection threshold of the RNAi screen. (DOC) [file pone.0035818.s006.doc]

**Table S2:**

| **Gene Symbol** | **Accession** | **MAD Score** |
| --- | --- | --- |
| RHOB | NM_004040 | -1.1061667 |
| PJA2 | NM_014819 | -0.7788114 |
| GNAS | NM_000516 | -0.6975453 |
| GLRA1 | NM_000171 | -0.5343517 |
| PPP1CA | NM_002708 | -0.4058559 |
| GPR61 | NM_031936 | -0.152461 |
| NTRK2 | NM_006180 | 0.0389725 |
| RASGRF1 | NM_002891 | 0.173086 |
| SFRS1 | NM_006924 | 0.204299 |
| NEUROD2 | NM_006160 | 0.5880406 |
